# Dexmedetomidine alleviates lung ischemia-reperfusion injury by inhibiting cuproptosis: an *in vivo* study

**DOI:** 10.3389/fphar.2025.1562535

**Published:** 2025-04-01

**Authors:** Hanqun Liu, Zicheng Wang, Tong Qiu, Wenxing Du, Zhe Wu, Sheng Chen, Wenjie Jiao

**Affiliations:** ^1^ Department of Thoracic Surgery, Affiliated Hospital of Qingdao University, Qingdao, Shandong, China; ^2^ Department of Thoracic Surgery, Peking University People’s Hospital, Beijing, Beijing, China

**Keywords:** lung ischemia-reperfusion injury, dexmedetomidine, cuproptosis, rats, *in vitro* experiments

## Abstract

Lung ischemia-reperfusion (I/R) injury represents an inevitable complication in lung transplantation, characterized by the excessive production of oxygen free radicals and toxic substances. Dexmedetomidine (DEX), a widely used anesthetic agent, has been shown to significantly elevate glutathione (GSH) levels, thereby conferring protection against copper influx. This study investigates the protective mechanisms of DEX in lung I/R injury, with a particular focus on cuproptosis. Utilizing a rat I/R model established by clamping the left hilum of lung for 90 min followed by 120 min of reperfusion, we examined the effects of DEX on lung injury scores, GSH content, and the expression of key proteins involved in cuproptosis. In conclusion, cuproptosis is implicated in pulmonary I/R injury, and the protective effect of DEX against lung I/R injury is partly mediated by inhibition of cuproptosis.

## 1 Introduction

Lung ischemia-reperfusion (I/R) injury represents a critical complication in thoracic surgeries, including lung transplantation and sleeve pulmonary lobectomy. This pathophysiological process is characterized by excessive generation of oxygen free radicals and cytotoxic metabolites (Ward, 1994), frequently resulting in postoperative pulmonary dysfunction and graft failure. Despite its clinical significance, the precise molecular mechanisms underlying lung I/R injury remain incompletely elucidated. Current research priorities focus on mechanistic exploration and therapeutic development to improve surgical outcomes.

Copper, an essential trace element, serves as a catalytic cofactor in redox reactions and regulates multiple enzymatic systems (Steffens et al., 1987; Harris, 1992; Smith-Mungo and Kagan, 1998; Ash et al., 1984). Beyond its established roles in hemoglobin synthesis, bone formation (Rondanelli et al., 2021), and neuroimmune homeostasis (Gromadzka et al., 2020), emerging evidence implicates copper dysregulation in cellular pathology. Mechanistically, impaired copper metabolism induces proteotoxic stress through ferredoxin 1 (FDX1)-mediated lipoylated protein aggregation, mitochondrial respiratory dysfunction, and depletion of iron-sulfur cluster proteins - a copper-dependent cell death pathway termed cuproptosis (Tsvetkov et al., 2022). This novel regulated cell death modality has been implicated in both chronic conditions (e.g., Wilson’s disease) and acute pathologies, including traumatic spinal cord injury (Li et al., 2023).

Dexmedetomidine (DEX), a selective α2-adrenergic receptor agonist with clinical applications in perioperative sedation, has demonstrated protective effects against pulmonary injury. Preclinical studies revealed its capacity to attenuate oxidative damage through ROS suppression and lipid peroxidation reduction (Fu et al., 2017). Specifically, Zhou et al. (Zhou et al., 2018)documented DEX-mediated mitigation of pulmonary I/R injury in rodent models via modulation of oxidative stress markers (MDA reduction and SOD elevation). While these antioxidant properties are well-documented, the molecular mechanisms underlying DEX’s pulmonary protection remain incompletely characterized.

This study investigates the hypothesis that DEX alleviates lung I/R injury through cuproptosis regulation. Utilizing a rat I/R model, we systematically analyze expression profiles of cuproptosis-related proteins and glutathione-mediated antioxidant pathways. Our findings provide novel insights into cuproptosis pathophysiology in pulmonary I/R injury and establish a theoretical framework for developing targeted therapeutic strategies.

## 2 Methods and materials

### 2.1 Animals

Male Sprague-Dawley rats (7–8 weeks old; Pengyue, Jinan, China) were housed in a controlled environment with a 12-h light/dark cycle and *ad libitum* access to food and water. All animals underwent a 3-day acclimatization period prior to experimentation. Animal procedures were conducted in accordance with the ARRIVE (Animal Research: Reporting of *In Vivo* Experiments) guidelines and approved by the Institutional Animal Care and Use Committee of the Affiliated Hospital of Qingdao University [AHQU-MAL20230607LH].

### 2.2 Lung I/R injury model

Body temperature was maintained at 36.5°C–39.0°C using a heating blanket throughout the procedure. Meloxicam (5 mg/kg) was administered subcutaneously for preemptive analgesia 1 h prior to anesthesia induction. Rats were anesthetized via intraperitoneal injection of sodium pentobarbital (60 mg/kg). Following tracheal intubation through a midline cervical incision, mechanical ventilation (initial tidal volume: 10 mL/kg) was initiated using a rodent ventilator. A femoral venous catheter was placed for drug administration.

**FIGURE 1:**
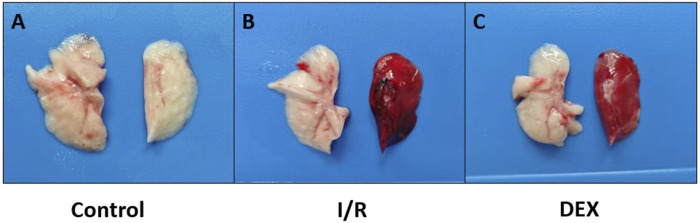
Photographs of rat lungs. Lungs were obtained immediately on ice blocks at the end of the experiment. **(A)** lungs in the control group; **(B)** lungs in the I/R group; **(C)** lungs in the DEX group. It was visualized that DEX reduced pulmonary congestion after lung ischemia-reperfusion.

After thoracotomy, heparin (300 IU/kg) was intravenously injected. Ten minutes post-heparinization, the left pulmonary hilum was occluded at end-expiration using a non-traumatic microvascular clamp. Lidocaine (20 mg) was topically applied to the surgical site for analgesia and phrenic nerve blockade. During ischemia, tidal volume was reduced to 6 mL/kg. Following 90 min of ischemia, clamps were removed and tidal volume restored to 10 mL/kg for 120-min reperfusion. Post-reperfusion, arterial blood was collected for gas analysis, and euthanasia was performed via exsanguination under deep anesthesia. Left lung tissues (Figure 1) and blood samples were harvested for subsequent analyses.

### 2.3 Experimental groups

Rats were randomly assigned to three groups (n = 6/group): Sham group: Thoracotomy without hilar clamping; I/R group: Full I/R protocol; DEX group: Dexmedetomidine (DEX; Sinopharm Chemical Reagent, 10 μg/kg) administered via femoral vein infusion over 20 min pre-thoracotomy, followed by I/R protocol.

### 2.4 Histopathological examination and lung injury scoring

Left lung lobes were fixed in 4% paraformaldehyde for 24 h, paraffin-embedded, and sectioned at 6 μm thickness for hematoxylin-eosin (H&E) staining. Histopathological evaluation was performed by a blinded pathologist using light microscopy. Lung injury scores (LIS) were calculated based on four parameters: (1) neutrophil infiltration, (2) interstitial edema, (3) hyaline membrane formation, and (4) hemorrhage. Each parameter was graded 0–4 (0 = normal, 1 = minimal, 2 = mild, 3 = moderate, 4 = severe).

### 2.5 Wet/dry (W/D) weight ratio

Surface moisture was carefully blotted from fresh lung tissues using filter paper. Wet weights were recorded before desiccation in a 75°C oven for 72 h. Dry weights were measured to calculate the W/D ratio.

### 2.6 Measurement of inflammatory factors

Blood samples were clotted at room temperature for 2 h, then centrifuged (3,000 × g, 15 min, 4°C) to collect serum. TNF-α, IL-1β, and IL-6 levels were quantified using commercial ELISA kits (Invitrogen) following manufacturer protocols.

### 2.7 Glutathione (GSH) assay

Lung tissues were homogenized in cold PBS and centrifuged (12,000 × g, 15 min, 4°C). Supernatants were analyzed using GSH assay kits (Elabscience, Wuhan, China). Absorbance at 450 nm was measured for both tissue and serum samples.

### 2.8 Western blotting

Lung tissues were lysed in RIPA buffer (Elabscience, Wuhan, China) supplemented with protease inhibitors, then sonicated (Sonic Dismembrator, United States). For lipoylated protein analysis, 10 μM TCEP was added prior to 95°C denaturation. Protein concentrations were determined via BCA assay (Elabscience), with equal amounts loaded onto 4%–20% gradient SDS-PAGE gels. Proteins were transferred to PVDF membranes and blocked with 5% non-fat milk. Membranes were incubated overnight at 4°C with primary antibodies against: lipoic acid (Abcam, 1:1,000), FDX1 (Abcam, 1:1,000), LIAS (Proteintech, 1:1,000), SDHB (Proteintech, 1:1,000), DLAT (Proteintech, 1:1,000), DLD (Proteintech, 1:1,000), SLC31A1 (MCE, 1:1,000), DLST (MCE, 1:1,000), and β-actin (Sevier, 1:5,000). After PBST washes, membranes were incubated with HRP-conjugated secondary antibodies (Proteintech, 1:10,000) for 1 h at room temperature. Protein bands were visualized using ECL reagent (Elabscience) and quantified with ImageJ software.

### 2.9 DLAT oligomerization analysis

Protein samples were prepared with non-reducing loading buffer (Beyotime, China) and minimally heated (70°C, 5 min). Electrophoresis and subsequent procedures followed standard Western blot protocols.

### 2.10 Immunohistochemical staining

Deparaffinized sections underwent antigen retrieval in citrate buffer (pH 6.0). Endogenous peroxidase was quenched with 3% H_2_O_2_, followed by blocking with 10% goat serum. Sections were incubated with anti-lipoic acid antibody (Abcam, 1:100) overnight at 4°C, then with HRP-conjugated secondary antibody (37°C, 1 h). Diaminobenzidine (DAB) was used for chromogenic detection. Hematoxylin staining was used to visualize the nuclei.

### 2.11 Immunofluorescence

After antigen retrieval and blocking, sections were incubated with anti-FDX1 (Abcam, 1:200) and anti-DLAT (Proteintech, 1:500) antibodies overnight at 4°C. Alexa Fluor-conjugated secondary antibodies (Invitrogen, 1:500) were applied for 1 h at room temperature. Nuclei were counterstained with DAPI (5 μg/mL). Images were acquired using an Olympus BX53 fluorescence microscope.

### 2.12 Statistical analysis

Data are presented as mean ± SD. Normality was assessed using Shapiro-Wilk test. One-way ANOVA with Tukey’s *post hoc* test was performed using Prism 10.1.2 (GraphPad). Statistical significance was set at p < 0.05.

## 3 Results

### 3.1 DEX attenuated histopathological damage

**FIGURE 2:**
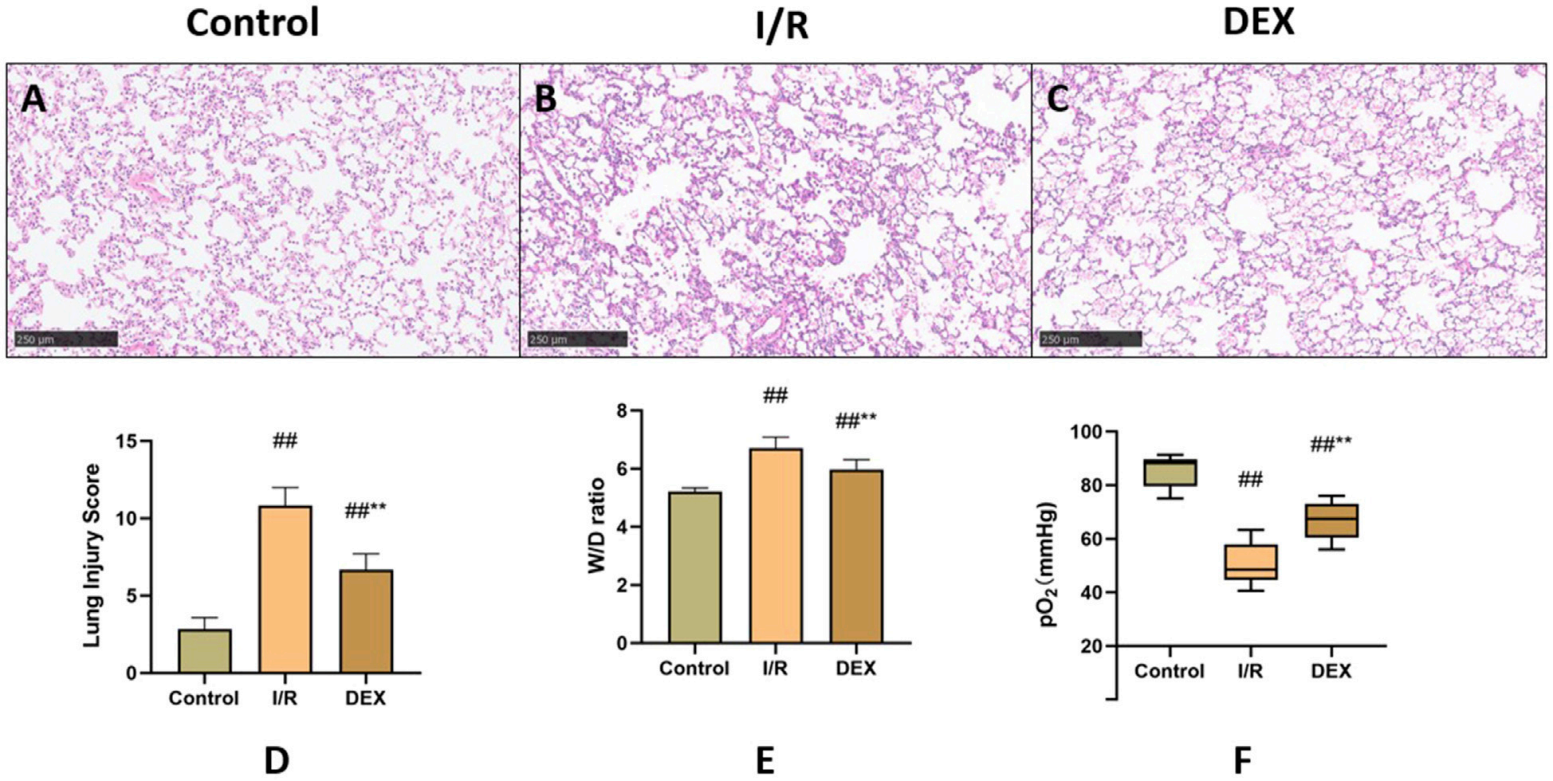
Comparison of lung tissue injury. **(A–C)** microscopic images stained with HE. **(D)** Lung injury score (n = 6). **(E)** lung tissue dry-wet weight ratio (n = 6). **(F)** rats’ blood gas parameters (n = 6). ## p < 0.05 vs. Control group; ** p < 0.05 vs. I/R group.

Histopathological analysis revealed distinct morphological changes among groups (Figures 2A–C). Compared to the sham control, the I/R group demonstrated significantly exacerbated neutrophilic infiltration, interstitial edema, and alveolar hemorrhage. DEX pretreatment markedly improved these pathological alterations. Semi-quantitative assessment of lung injury scores (Figure 2D) confirmed progressive deterioration from control (minimal injury) to I/R groups (severe injury), with DEX intervention showing intermediate protection. The W/D ratio analysis (Figure 2E) paralleled these findings, showing significant elevation in I/R group compared to control, which was effectively reduced by DEX administration (p = 0.0059 vs. I/R).

### 3.2 DEX improved oxygenation parameters

Arterial blood gas analysis (Figure 2F) indicated that lung I/R injury significantly compromised oxygen exchange capacity, as evidenced by decreased PaO_2_ levels. DEX treatment partially restored oxygenation parameters, showing statistically significant improvement compared to I/R group (p = 0.004).

### 3.3 DEX suppressed systemic inflammatory response

**FIGURE 3:**
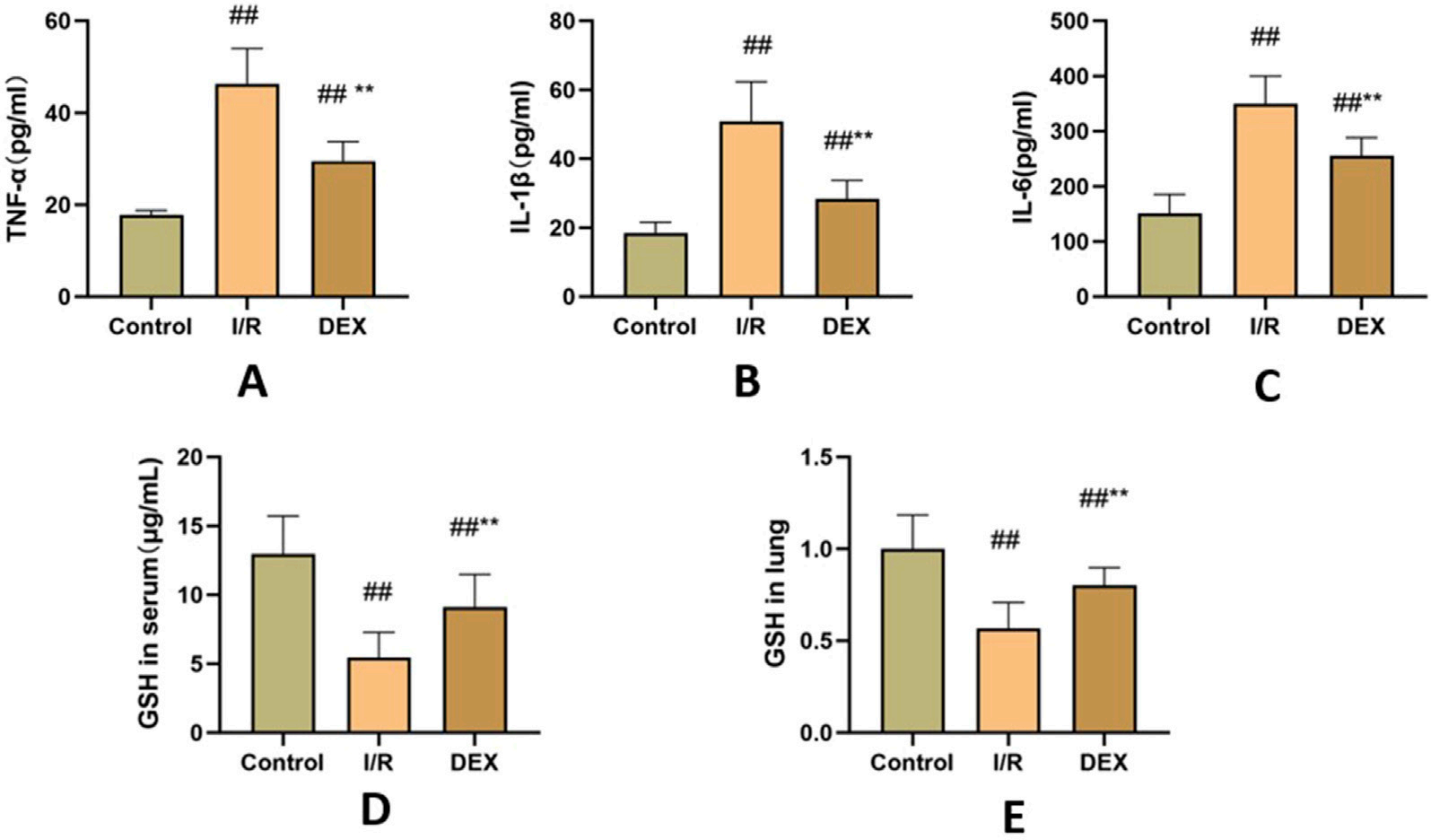
Inflammatory factors and GSH. **(A)** concentrations of TNF-α(n = 6). **(B)** concentrations of IL-1β(n = 6). **(C)** concentrations of IL-6 (n = 6). **(D)** concentrations of GSH in serum. **(E)** the ratio of GSH content in lung tissue. ## p < 0.05 vs. control group; ** p < 0.05 vs. I/R group.

The I/R procedure induced significant elevation of serum pro-inflammatory cytokines (Figures 3A–C). TNF-α, IL-1β, and IL-6 levels were markedly increased in I/R group versus control (p < 0.05 for all comparisons). DEX pretreatment substantially mitigated this inflammatory cascade, demonstrating significant reduction in all measured cytokines compared to I/R group (p < 0.05).

### 3.4 DEX enhanced antioxidant capacity

As shown in Figures 3D, E, I/R injury caused significant depletion of GSH content in both lung tissue and serum compared to control group (p < 0.05). DEX administration effectively reversed this trend, restoring GSH levels to values significantly higher than those observed in I/R group (p < 0.05).

### 3.5 DEX modulated cuproptosis-associated proteins

**FIGURE 4:**
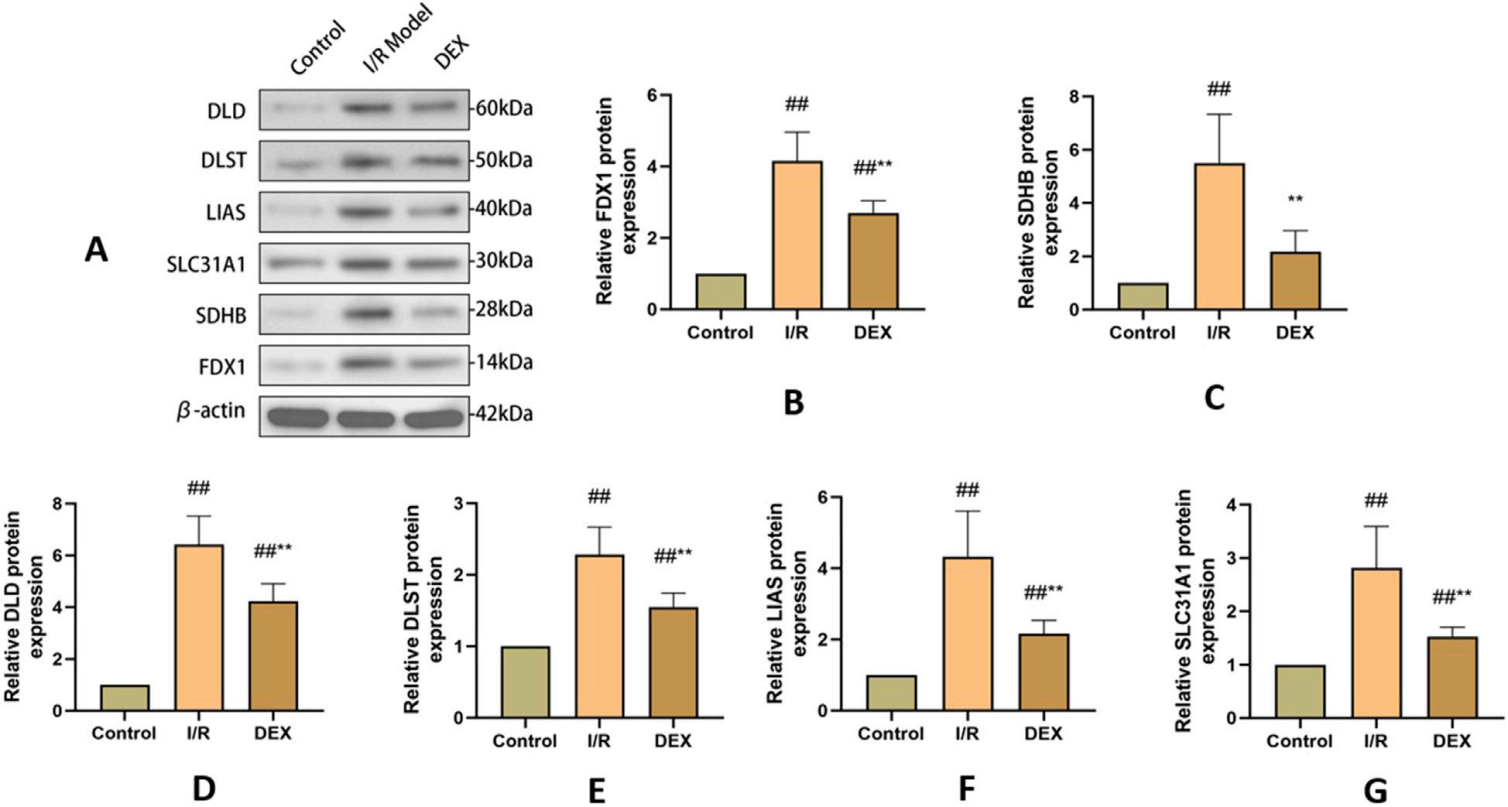
WB results of cuproptosis-related protein expression. **(A)** Western blots for FDX 1, SLC 31 A1, LIAS, DLST, DLD, and SDHB in rat lung tissue. **(B–G)** relative levels of FDX 1, SLC 31 A1, LIAS, DLST, DLD, and SDHB in rat lung tissue (n = 3). ## p < 0.05 vs. control group; ** p < 0.05 vs. I/R group.

**FIGURE 5:**
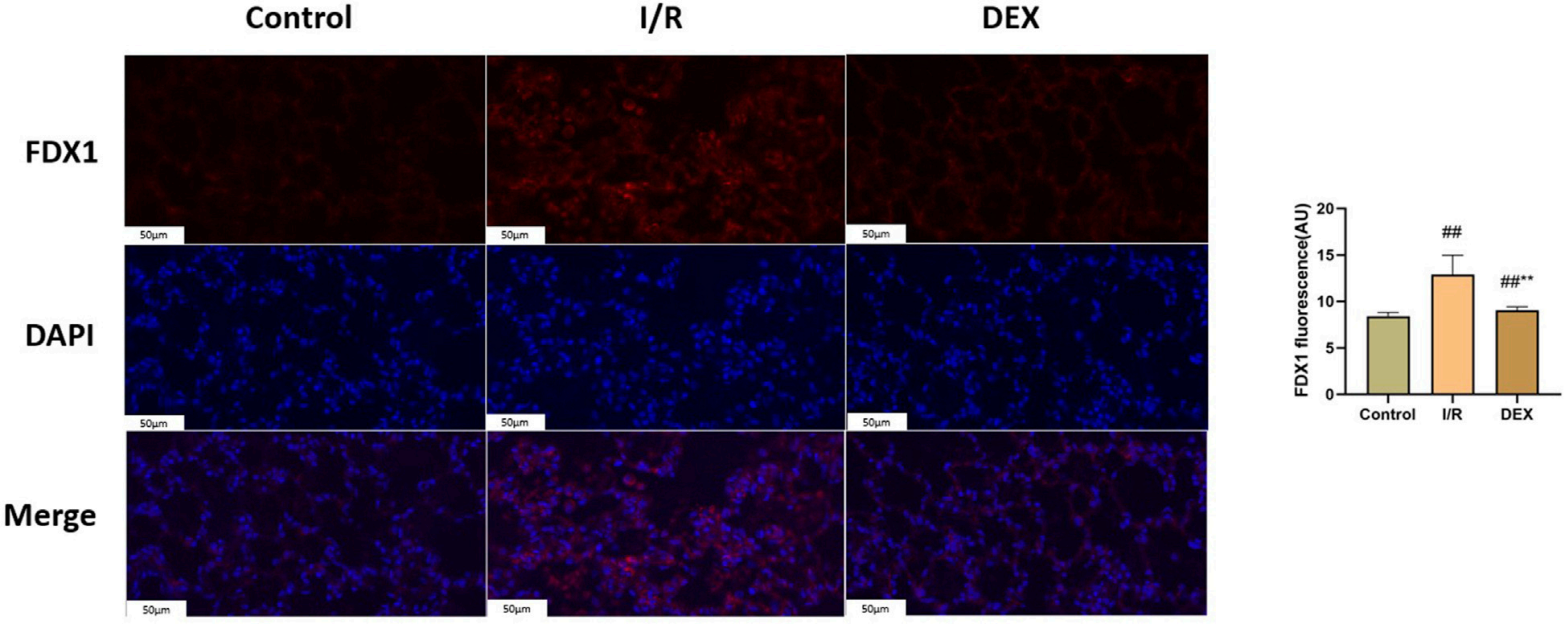
Immunofluorescence results of FDX1. Representative images of fluorescence probe for FDX1 and statistical comparison (n = 6). ## p < 0.05 vs. control group; ** p < 0.05 vs. I/R group.

Western blot analysis demonstrated I/R-induced upregulation of key cuproptosis regulators including FDX1, SLC31A1, LIAS, DLST, DLD, and SDHB (Figure 4). DEX treatment significantly attenuated these protein level alterations compared to I/R group (p < 0.05). Immunofluorescence quantification confirmed increased FDX1 expression in I/R lungs, which was reduced by DEX intervention (Figure 5).

### 3.6 DEX inhibited lipoylated protein accumulation

**FIGURE 6:**
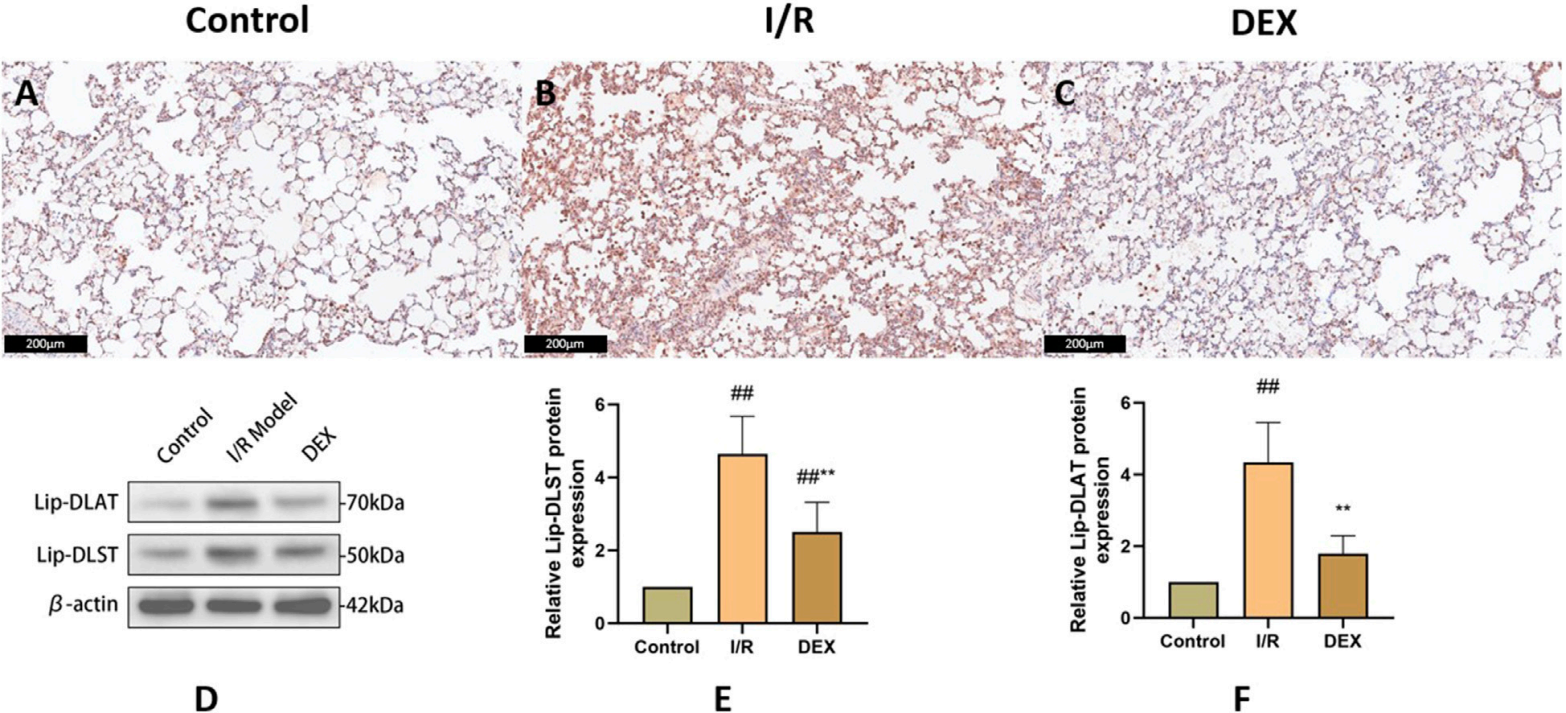
Immunohistochemical and WB results of lipoacylated proteins. **(A–C)** representative images of lipoacylated protein immunohistochemical staining in rat lung tissue. **(D)** Western blots for lip-DLAT and lip-DLST. **(E, F)** relative levels of lip-DLAT and lip-DLST in rats lung tissue (n = 3). ## p < 0.05 vs. control group; ** p < 0.05 vs. I/R group.

**FIGURE 7:**
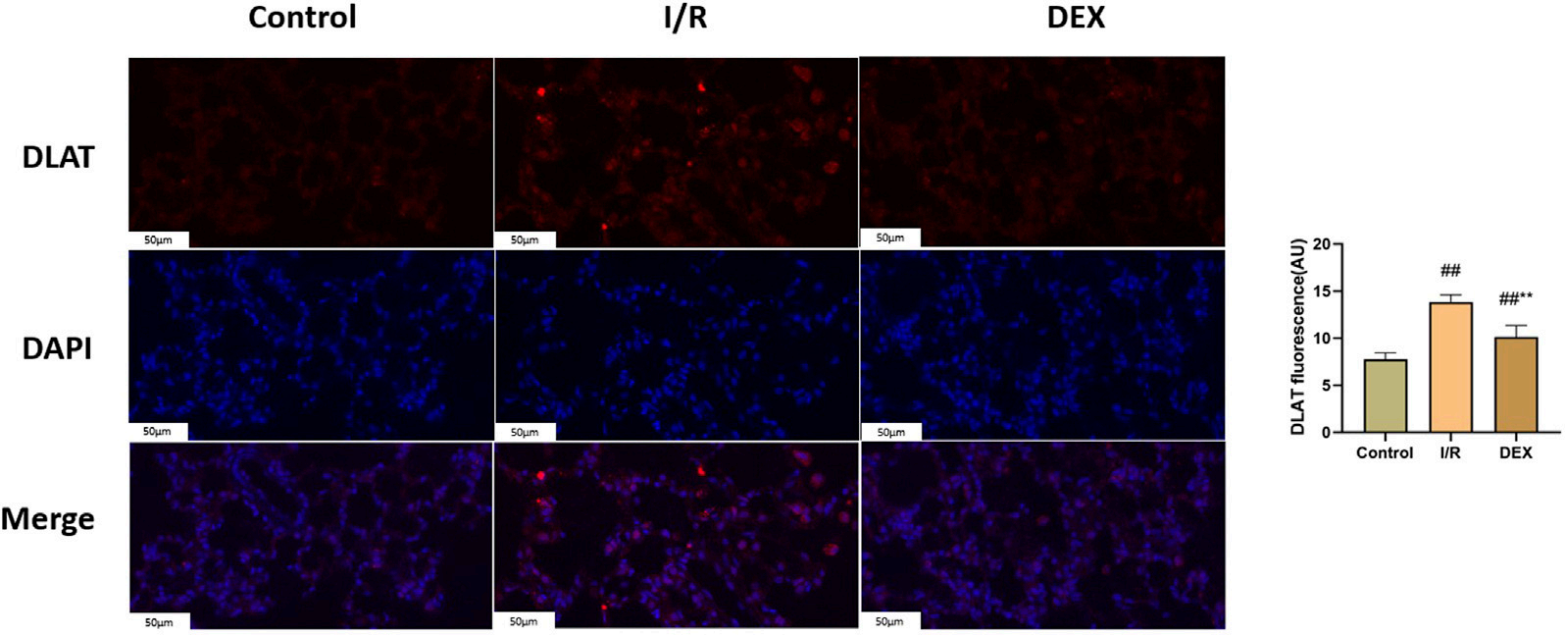
Immunofluorescence results of DLAT. Representative images of fluorescence probe for DLAT and statistical comparison (n = 6). ## p < 0.05 vs. control group; ** p < 0.05 vs. I/R group.

**FIGURE 8:**
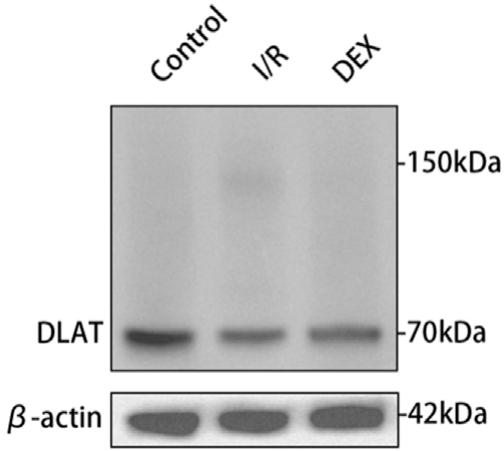
Oligomerization of the DLAT protein in WB experiment. DLAT protein oligomerization was observed in the I/R group, but not in the other two groups.

Immunoblot and immunohistochemical analyses revealed significant I/R-induced accumulation of lipoylated DLAT and DLST (Figure 6). DEX administration significantly reduced lipoylated protein levels compared to I/R group (p < 0.05). Non-reducing Western blot demonstrated prominent DLAT oligomerization in I/R group, which was effectively suppressed by DEX treatment (Figure 7). Immunofluorescence analysis corroborated these findings, showing significant reduction of DLAT aggregates with DEX intervention (Figure 8).

## 4 Discussion

Our study demonstrates that dexmedetomidine (DEX) administration effectively attenuates lung ischemia-reperfusion (I/R) injury, and this process is accompanied by inhibition of the cuproptosis. The therapeutic effects manifested as improved oxygenation parameters, reduced histopathological damage, and suppression of pro-inflammatory cytokine release.

Copper homeostasis plays pivotal roles in physiological processes, requiring precise regulation through an integrated network of copper-dependent proteins including transporters, chaperones, and enzymes (Zhong et al., 2022; Cobine and Brady, 2022; Bharathi Devi et al., 2016). Central to this regulatory system is solute carrier family 31 member 1 (SLC31A1), the principal copper importer governing cellular copper influx (Qi et al., 2023; Das et al., 2022; Xie et al., 2023). Our findings reveal that DEX significantly downregulates SLC31A1 expression, suggesting direct interference with copper uptake mechanisms. This observation aligns with computational modeling data indicating potential molecular interactions between DEX and SLC31A1, though experimental validation of this binding specificity requires further investigation.

The antioxidant glutathione (GSH) emerged as another critical mediator in DEX’s protective mechanism. We observed that DEX administration counteracts I/R-induced GSH depletion in both pulmonary tissue and systemic circulation. Given GSH’s established role in copper chelation and detoxification (Saporito-Magriñá et al., 2018), this restoration likely disrupts copper-mediated oxidative cascades. Notably, the coordinated downregulation of SLC31A1 and upregulation of GSH suggests synergistic modulation of copper homeostasis, potentially through adrenergic receptor-dependent signaling pathways warranting further exploration.

Ferredoxin 1 (FDX1), a key reductase facilitating copper redox cycling and protein lipoylation (Tsvetkov et al., 2022), displayed marked overexpression in I/R lungs that was attenuated by DEX treatment. The FDX1 suppression correlated with reduced lipoylated protein accumulation (DLAT/DLST) and diminished DLAT oligomerization - hallmark features of cuproptosis. FDX one results in oxidative stress inside the cell. Oxygen free radicals have been demonstrated to significantly contribute to cell death in organ I/R (Sadrkhanloo et al., 2022). Mechanistically, FDX1 inhibition may preserve cellular redox balance by maintaining GSH/GSSG ratios critical for free radical scavenging (Chen et al., 2022; Poprac et al., 2017). This dual regulation of copper metabolism and oxidative stress pathways positions FDX1 as a crucial nexus in DEX-mediated protection against I/R injury. DEX significantly inhibited FDX1/SLC31A1 expression, reduced copper influx, and restored TCA cycle function. This process may regulate protein post-translational modification through the α2-adrenoceptor mediated PI3K/Akt/mTOR signaling pathway (Zhao et al., 2020; Huang et al., 2024).

Our study has some limitations. First, the absence of cellular models limits mechanistic depth. Genetic manipulation (FDX1 knockout/overexpression) *in vitro* could clarify its regulatory hierarchy and enable mitochondrial functional analyses. Second, the lack of established cuproptosis inhibitors as positive controls prevents direct comparison of DEX’s therapeutic efficacy. Future studies should incorporate pharmacological (e.g., tetrathiomolybdate) and genetic interventions to validate target specificity.

## 5 Conclusion

There is a cuproptosis mechanism in the process of lung ischemia-reperfusion. The protective effect of DEX against lung I/R injury is partly mediated by inhibition of cuproptosis.

## Data Availability

The original contributions presented in the study are included in the article/Supplementary Material, further inquiries can be directed to the corresponding author.
