# Supplementary material for: Dexmedetomidine alleviates lung ischemia-reperfusion injury by inhibiting cuproptosis: an in vivo study
Source: Front Pharmacol. 2025 Apr 1;16:1562535. doi: 10.3389/fphar.2025.1562535 (PMC11996775; doi:10.3389/fphar.2025.1562535)
Supplement: Supplementary file 1 [file Presentation1.pptx]

## Slide 1
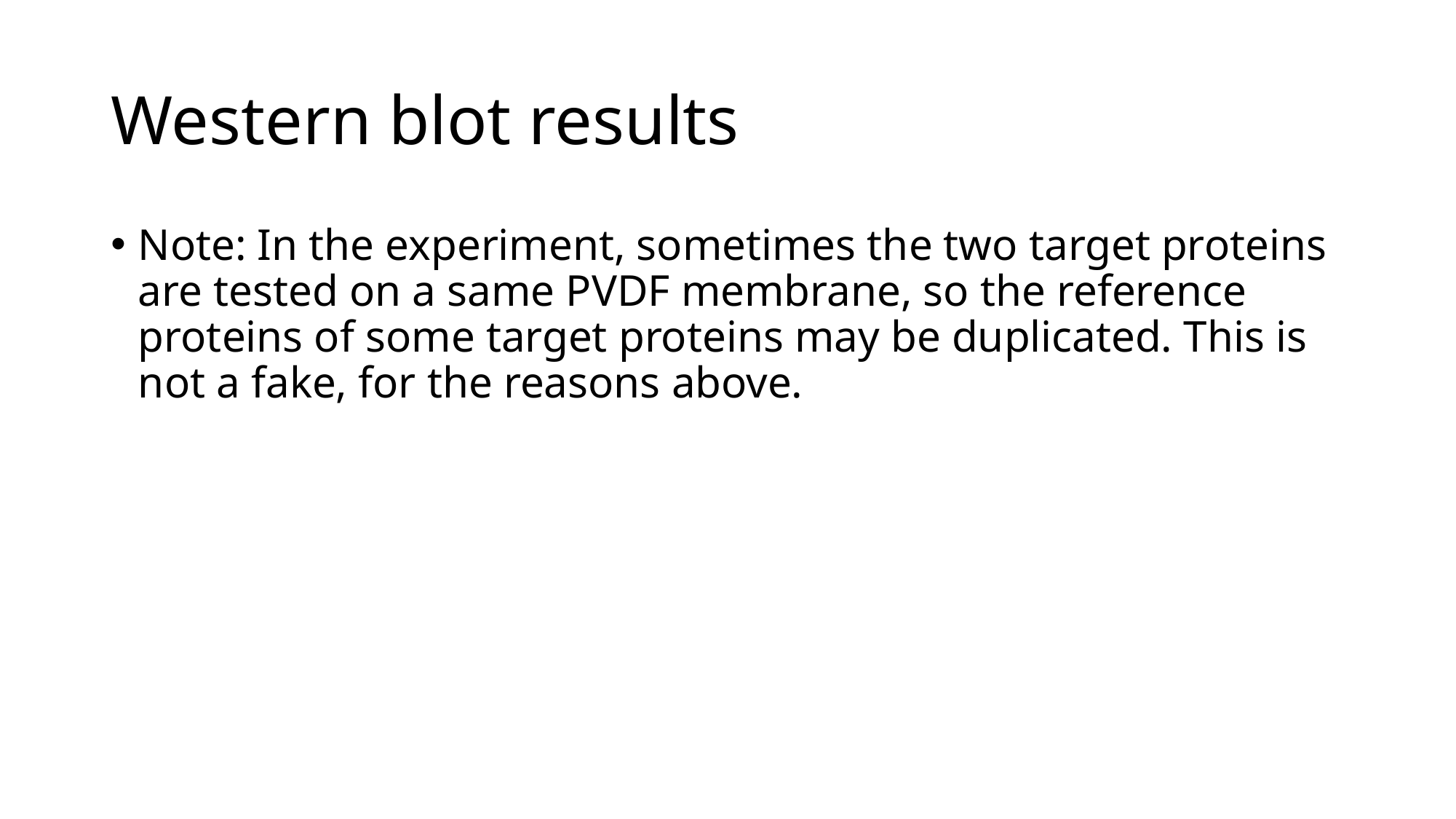

# Western blot results
Note: In the experiment, sometimes the two target proteins are tested on a same PVDF membrane, so the reference proteins of some target proteins may be duplicated. This is not a fake, for the reasons above.

## Slide 2
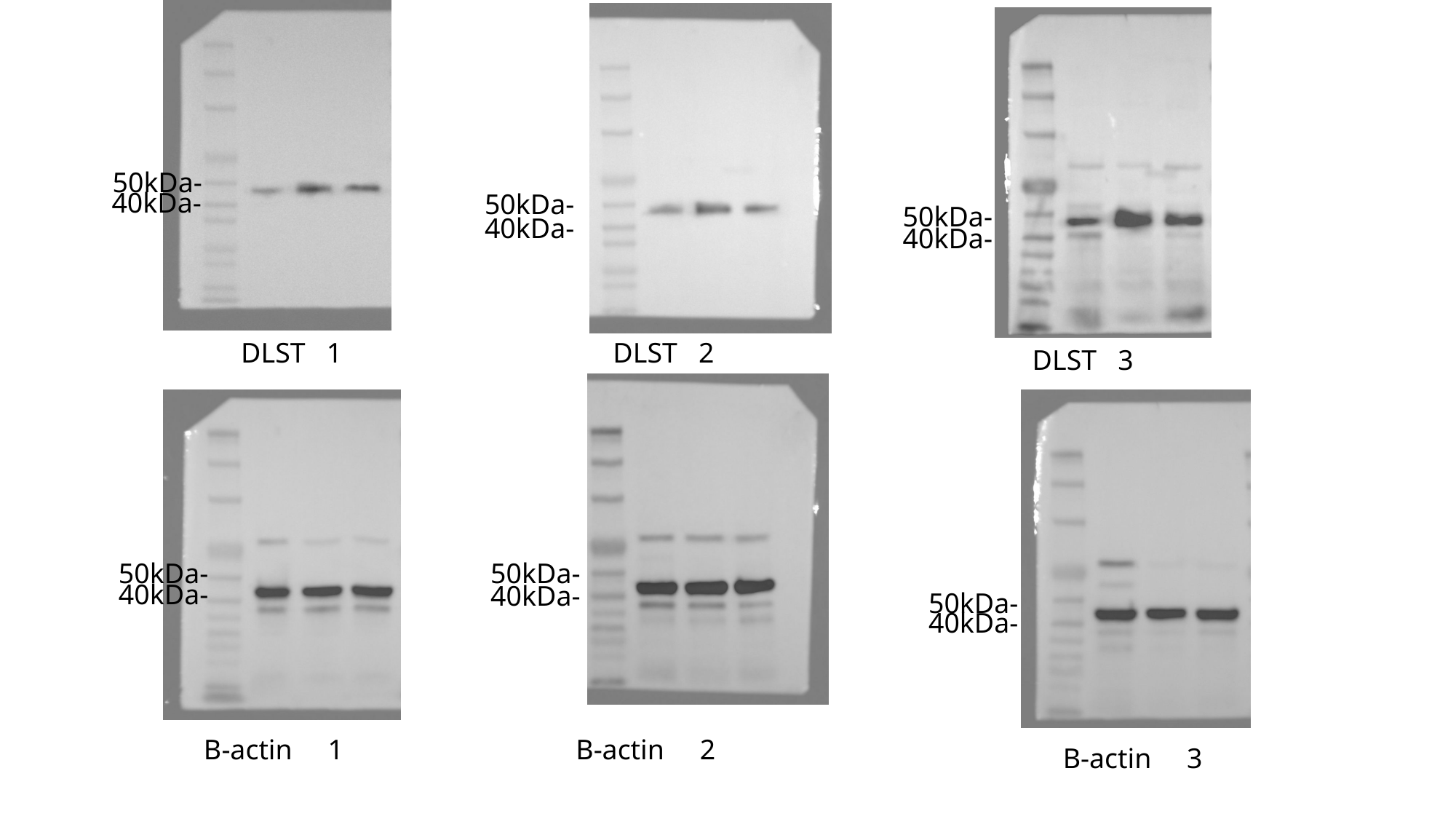

50kDa-
40kDa-
50kDa-
50kDa-
40kDa-
40kDa-
DLST 1
DLST 2
DLST 3
50kDa-
50kDa-
40kDa-
40kDa-
50kDa-
40kDa-
Β-actin 1
Β-actin 2
Β-actin 3

## Slide 3
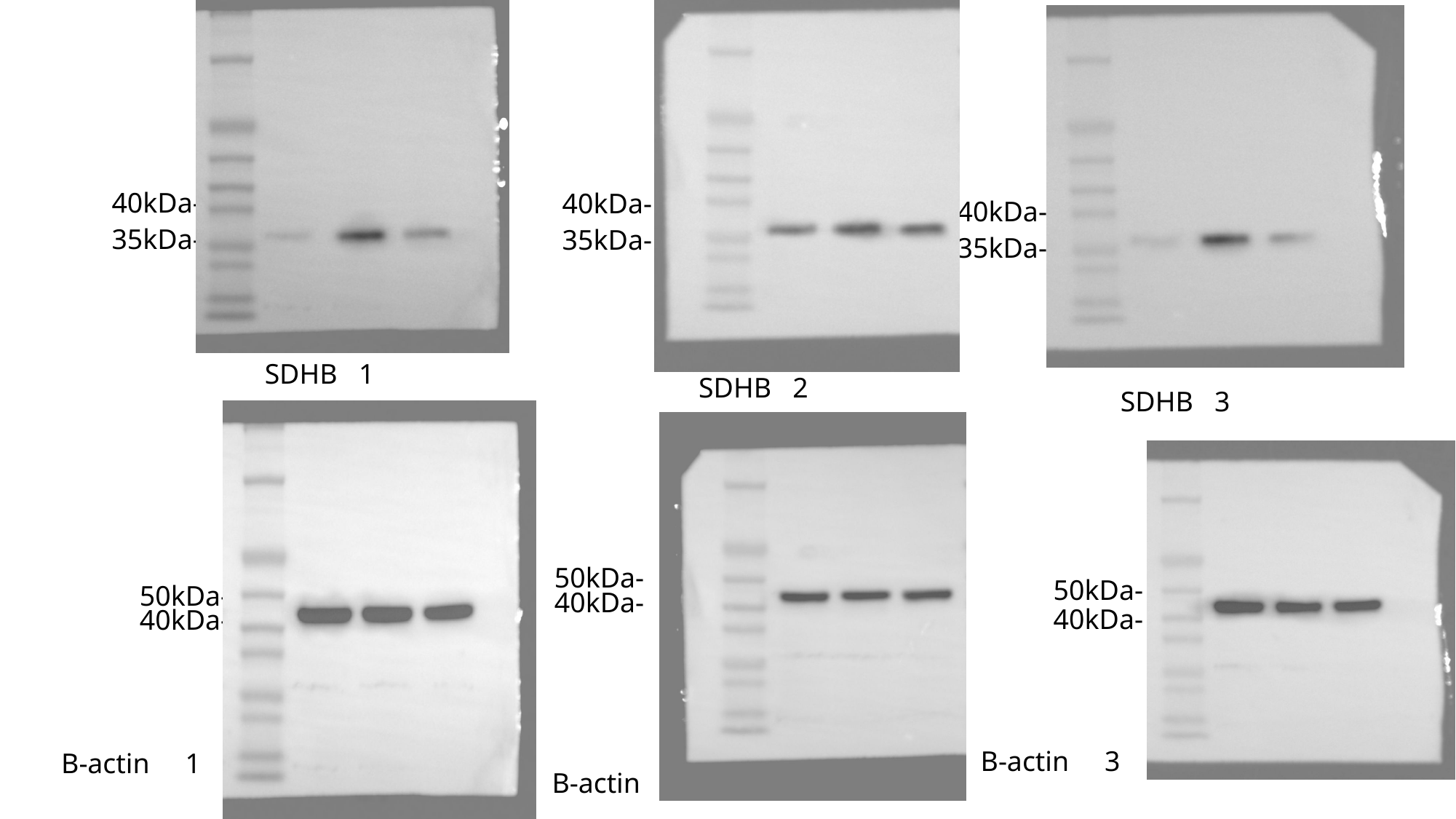

40kDa-
40kDa-
40kDa-
35kDa-
35kDa-
35kDa-
SDHB 1
SDHB 2
SDHB 3
50kDa-
50kDa-
50kDa-
40kDa-
40kDa-
40kDa-
Β-actin 3
Β-actin 1
Β-actin 2

## Slide 4
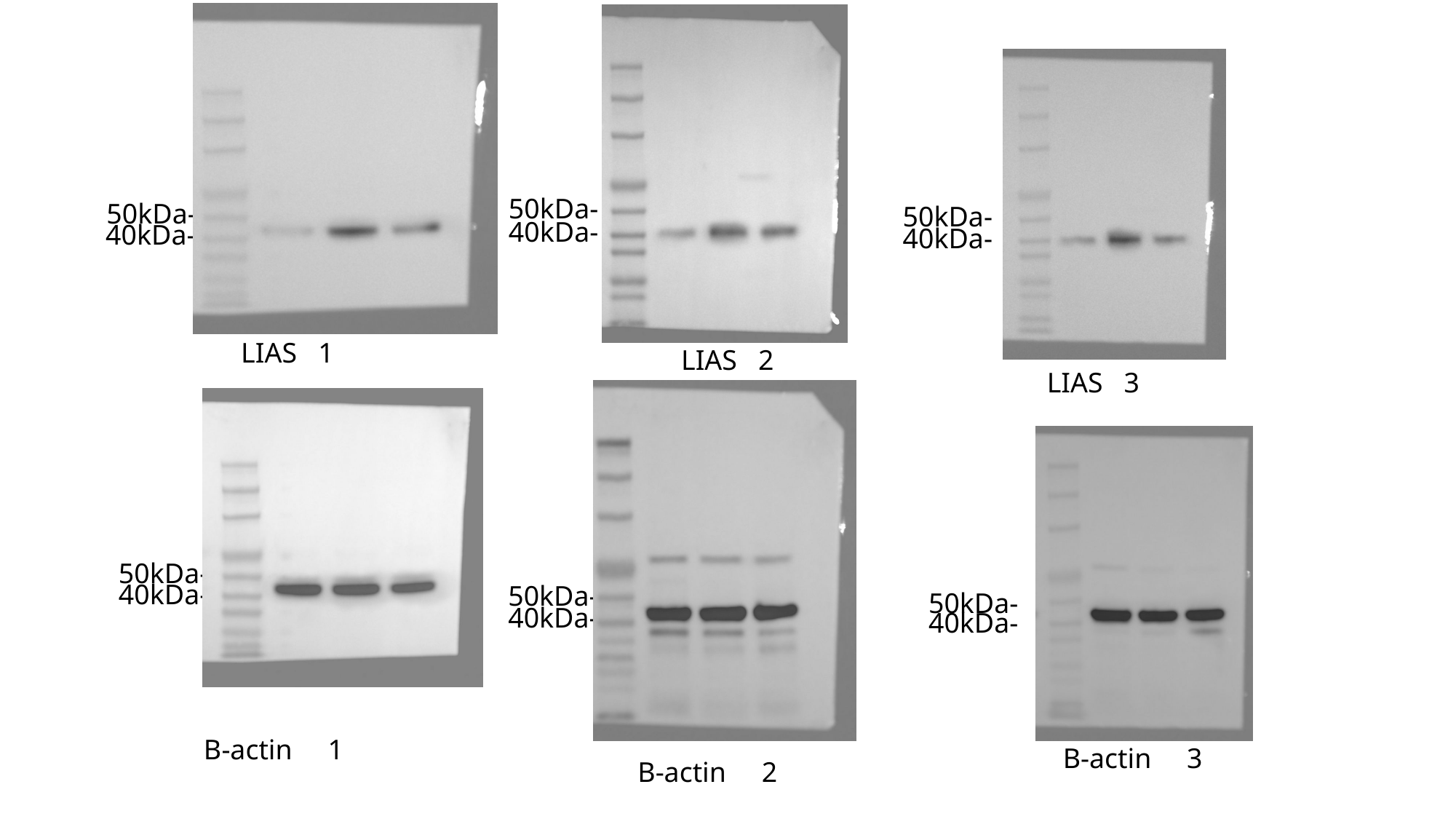

50kDa-
50kDa-
50kDa-
40kDa-
40kDa-
40kDa-
LIAS 1
LIAS 2
LIAS 3
50kDa-
40kDa-
50kDa-
50kDa-
40kDa-
40kDa-
Β-actin 1
Β-actin 3
Β-actin 2

## Slide 5
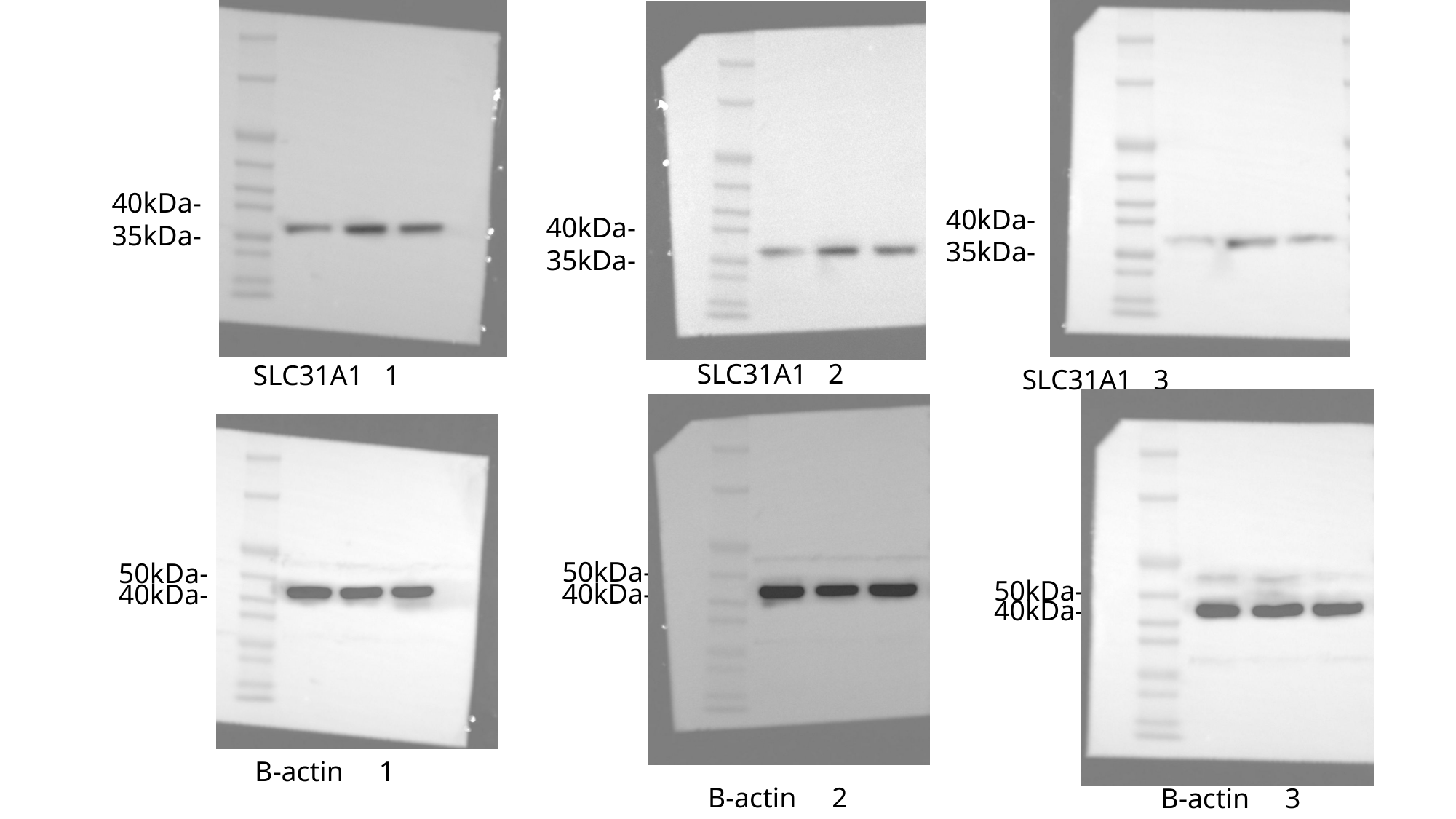

40kDa-
40kDa-
40kDa-
35kDa-
35kDa-
35kDa-
SLC31A1 2
SLC31A1 1
SLC31A1 3
50kDa-
50kDa-
50kDa-
40kDa-
40kDa-
40kDa-
Β-actin 1
Β-actin 2
Β-actin 3

## Slide 6
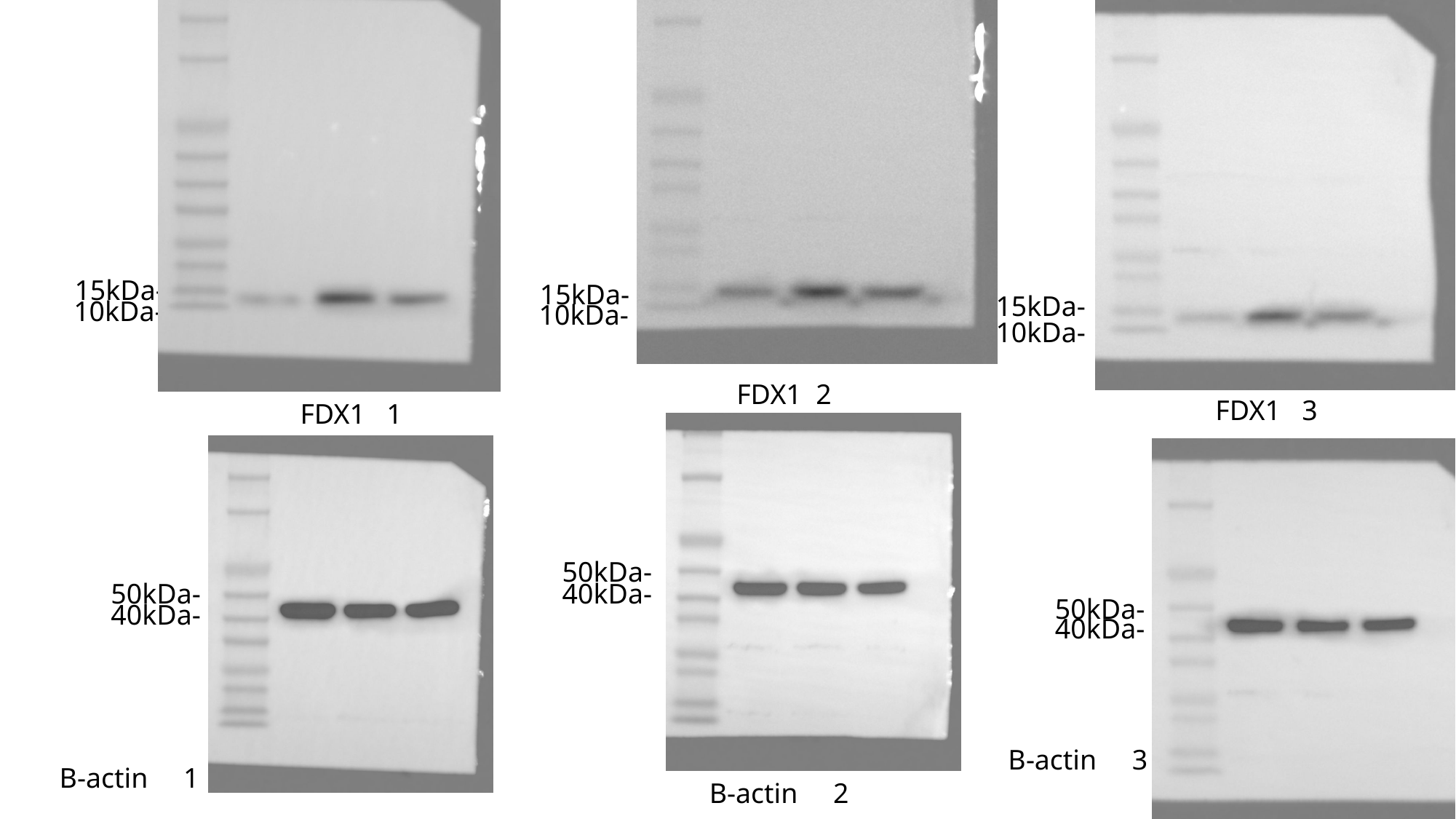

15kDa-
15kDa-
15kDa-
10kDa-
10kDa-
10kDa-
FDX1 2
FDX1 3
FDX1 1
50kDa-
50kDa-
40kDa-
50kDa-
40kDa-
40kDa-
Β-actin 3
Β-actin 1
Β-actin 2

## Slide 7
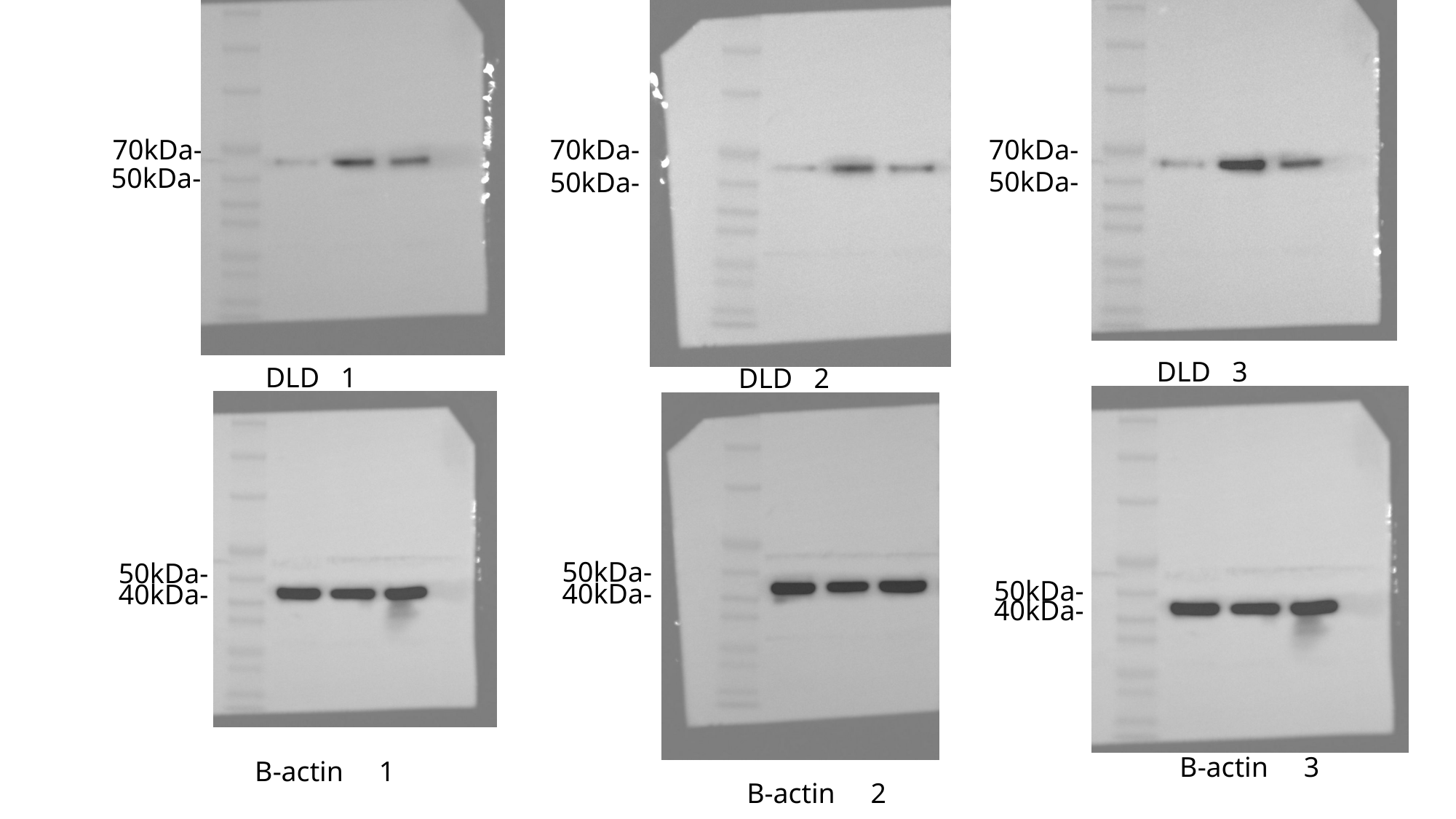

70kDa-
70kDa-
70kDa-
50kDa-
50kDa-
50kDa-
DLD 3
DLD 1
DLD 2
50kDa-
50kDa-
50kDa-
40kDa-
40kDa-
40kDa-
Β-actin 3
Β-actin 1
Β-actin 2

## Slide 8
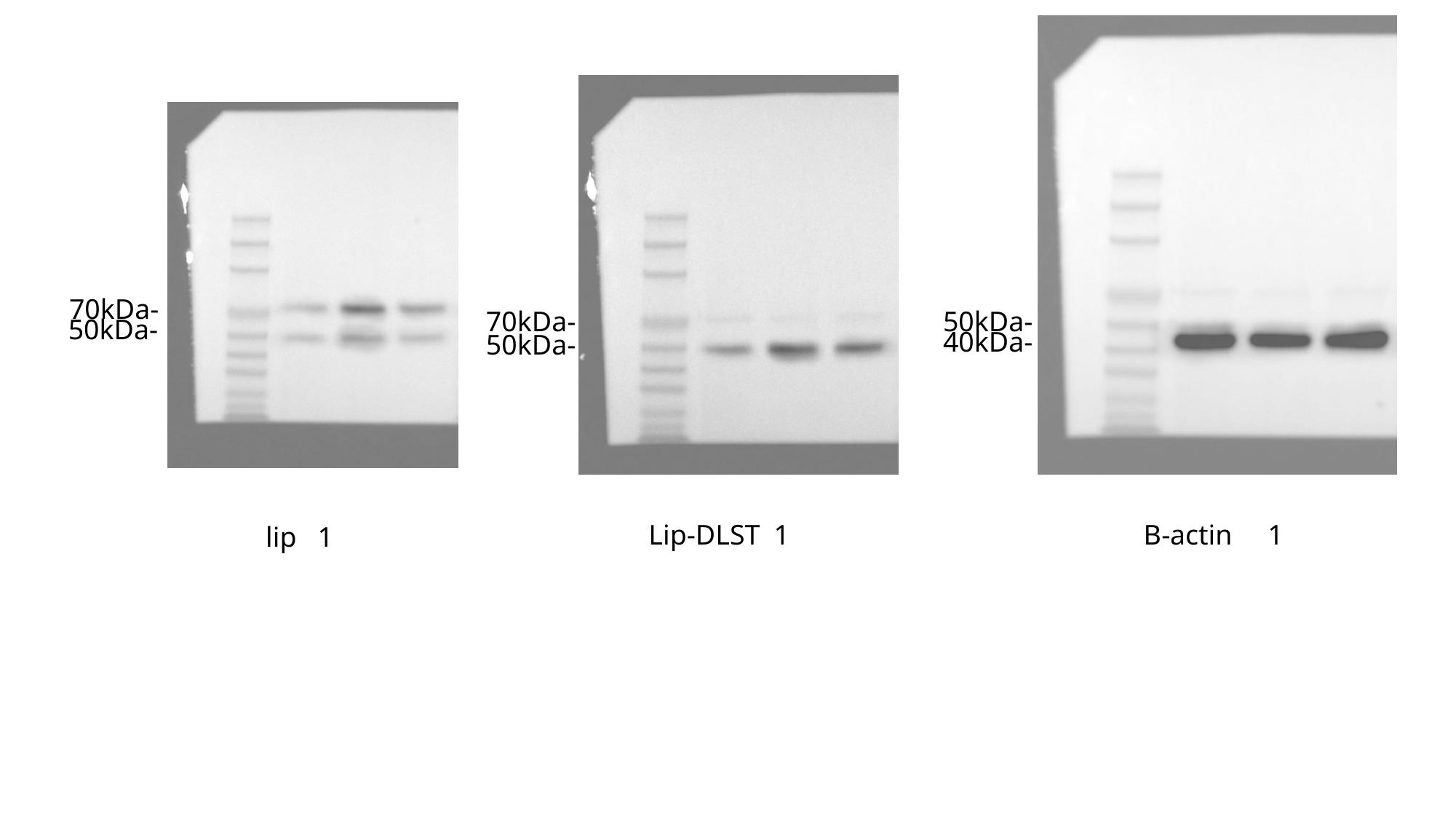

70kDa-
70kDa-
50kDa-
50kDa-
40kDa-
50kDa-
lip1
Lip-DLST 1
Β-actin 1
lip 1

## Slide 9
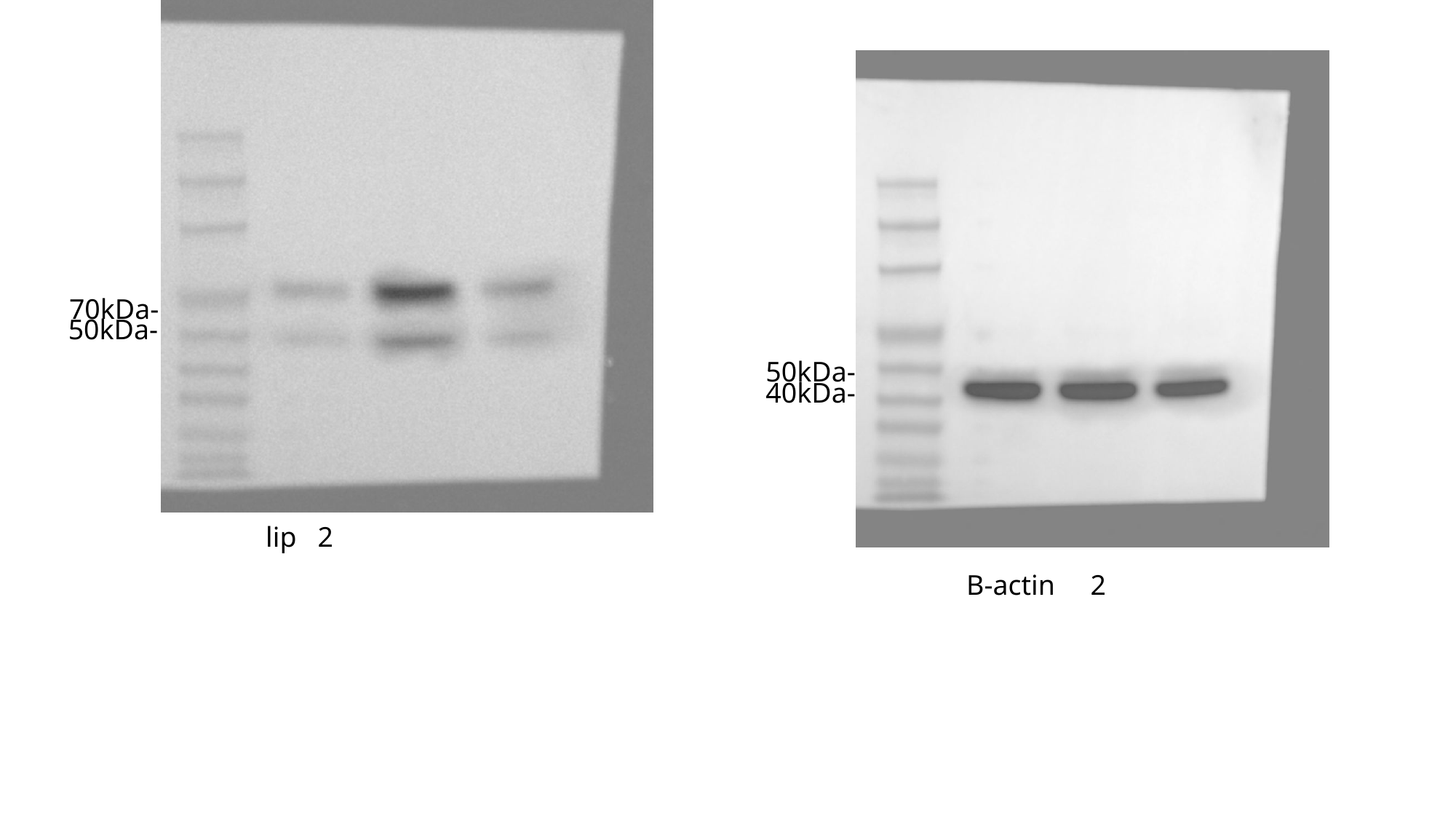

70kDa-
70kDa-
50kDa-
50kDa-
50kDa-
40kDa-
lip 2
Β-actin 2

## Slide 10
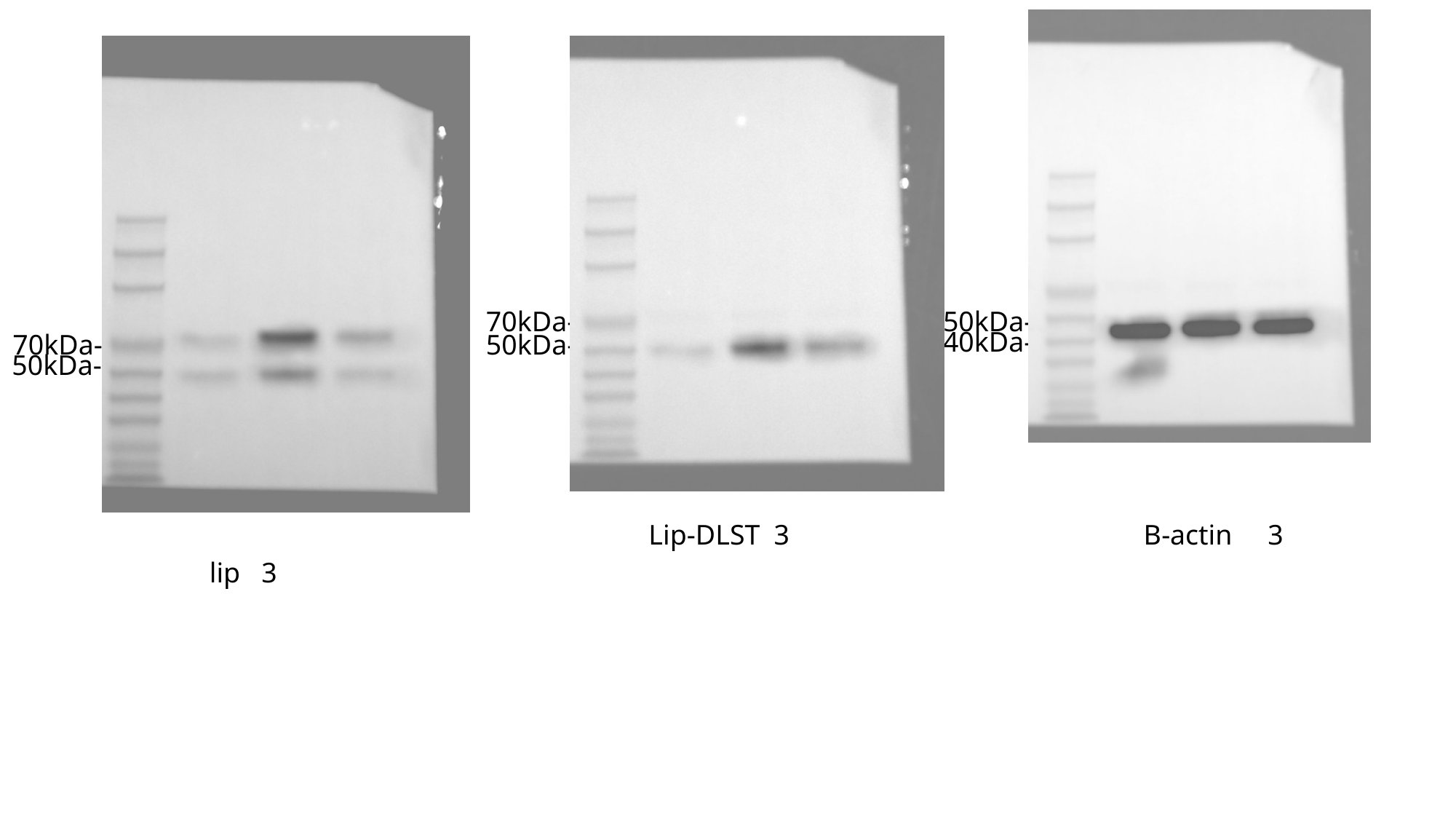

70kDa-
50kDa-
40kDa-
70kDa-
50kDa-
50kDa-
Lip-DLST 3
Β-actin 3
lip 3

## Slide 11
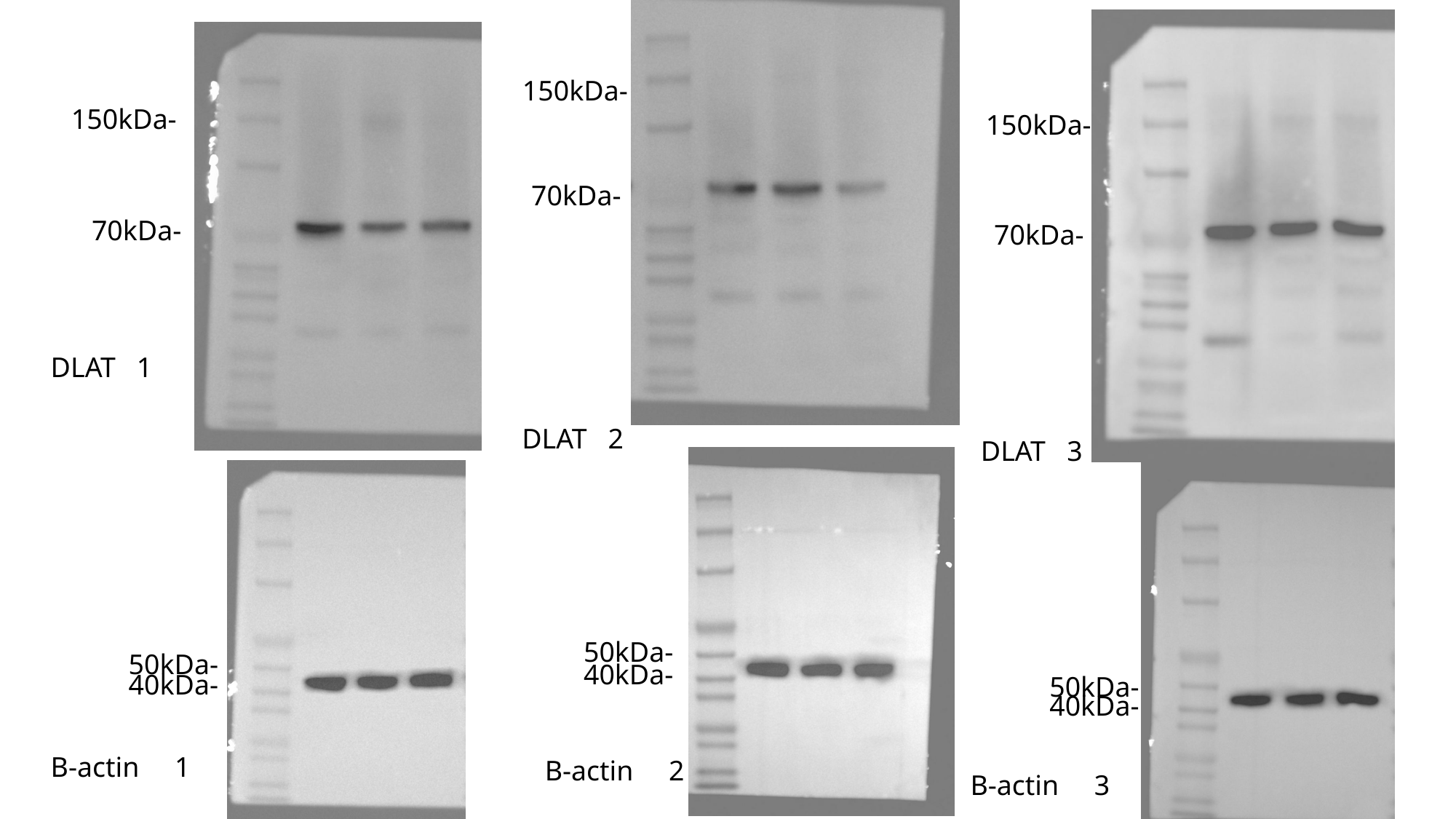

150kDa-
150kDa-
150kDa-
70kDa-
70kDa-
70kDa-
DLAT 1
DLAT 2
DLAT 3
50kDa-
50kDa-
40kDa-
40kDa-
50kDa-
40kDa-
Β-actin 1
Β-actin 2
Β-actin 3
